# Melanin and Neurotransmitter Signalling Genes Are Differentially Co‐Expressed in Growing Feathers of White and Rufous Barn Owls

**DOI:** 10.1111/pcmr.70001

**Published:** 2025-02-05

**Authors:** Anne‐Lyse Ducrest, Luis M. San‐Jose, Samuel Neuenschwander, Emanuel Schmid‐Siegert, Céline Simon, Marco Pagni, Christian Iseli, Hannes Richter, Nicolas Guex, Tristan Cumer, Emmanuel Beaudoing, Mélanie Dupasquier, Pauline Charruau, Pauline Ducouret, Ioannis Xenarios, Jérôme Goudet, Alexandre Roulin

**Affiliations:** ^1^ Department of Ecology and Evolution, Biophore University of Lausanne Lausanne Switzerland; ^2^ Centre de Recherche sur la Biodiversité et l'Environnement (CRBE) Université de Toulouse, CNRS, IRD, Toulouse INP, Université Toulouse 3 – Paul Sabatier (UT3) Toulouse France; ^3^ Vital‐IT, Swiss Institute of Bioinformatic Lausanne Switzerland; ^4^ Department of Computational Biology University of Lausanne Lausanne Switzerland; ^5^ JSR‐NGSAI Epalinges Switzerland; ^6^ Bioinformatics Competence Center University of Lausanne Lausanne Switzerland; ^7^ Bioinformatics Competence Center Ecole Polytechnique Fédérale de Lausanne Lausanne Switzerland; ^8^ Centre for Integrative Genomics, Genomic Technologies Facility University of Lausanne Lausanne Switzerland; ^9^ Agora Center Lausanne Switzerland; ^10^ Health2030 Genome Center Genève Switzerland

**Keywords:** barn owl (*Tyto alba*), melanin, melanocortin 1 receptor (MC1R), neurotransmitter‐related genes

## Abstract

Regulation of melanin‐based pigmentation is complex, involving multiple genes. Because different genes can contribute to the same pigmentation phenotype, the genes identified in model organisms may not necessarily apply to wild species. In the barn owl ( *Tyto alba* ), ventral plumage colour ranges from white to rufous, with genetic variation in the melanocortin 1 receptor gene (*MC1R*) accounting for at least a third of this variation. In the present study, we used transcriptomic data to compare the gene expression profiles of growing feathers from nestlings with different *MC1R* genotypes. We identified 21 differentially expressed genes, nine of which are involved in melanogenesis, while seven are related to neurotransmitter function or synaptic activity. With the exception of *CALB1*, all of the differentially expressed genes were upregulated in rufous owls compared to white barn owls. To the best of our knowledge, this study is the first to link melanin production with neurotransmitter‐related genes, and we discuss possible evolutionary explanations for this connection.

## Graphical abstract

The colouration of the ventral face of barn owls varies from white to rufous, depending on the MC1R (V126I) genotype. This colouration is linked to multiple fitness and behavioural traits. Genes linked to neurotransmitters and synapses are upregulated with melanin genes in a transcriptome of growing feathers from the ventral part of chicks of the MC1RVV and MC1RVI genotypes.

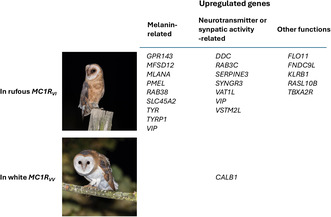

**Summary**

- The melanocortin 1 receptor (MC1R) regulates melanin synthesis, pain perception and anti‐inflammatory processes and may protect against Parkinson's disease.
- It is thus important to know which genes are regulated by MC1R.
- In this context, barn owls are interesting, as their ventral body side varies from white to reddish‐brown depending on their *MC1R* genotypes (*V126I*) and their colouration is related to multiple fitness and behavioural traits.
- A transcriptome of growing feathers showed that *MC1R* genotypes influence the expression of genes linked to neurotransmitters that are co‐expressed with melanin genes.
- We discuss the implications of this discovery.

## 1 Introduction

The observation that colouration varies so much between organisms has attracted the attention of a large range of researchers from evolutionary biologists interested in the adaptive function of colour patterns to molecular biologists who are interested in the underlying mechanisms of colour variation. In animals, different types of pigments, such as carotenoids, pterins, psittacofulvins, porphyrins and melanins, are responsible for such diversity. Melanin in particular is present in a large range of organisms from bacteria to invertebrates and vertebrates and plays a role in UV protection, thermoregulation, protection against abrasion and pathogens, camouflage and signalling (McNamara et al. 2021; San‐Jose and Roulin 2018). It produces colouration from reddish‐yellow brown (pheomelanin) to grey black (eumelanin) (Wakamatsu, Zippin, and Ito 2021) and its interaction with the structural components of the integument results in iridescent colours (Shawkey, Morehouse, and Vukusic 2009).

In vertebrates, melanin production takes place in lysosome‐like organelles, melanosomes, within specialised cells, melanocytes. Most of the melanocytes originate from the neural crest cells and migrate to the skin (Vandamme and Berx 2019). In birds, melanocytes are mainly distributed in the basal layer of the skin epidermis and in feather follicles in which melanocyte progenitor cells reside in the dermal papilla; a persistent structure at the base of the mesenchyme from which feathers are generated (Wu et al. 2021). At hatching, chicks already possess feather follicles that produce down feathers, which are replaced a few weeks later by nestling feathers and maintained throughout their lifetime by multiple cycles of moulting and feather regeneration (Chen et al. 2015).

As a feather grows, the follicle enlarges and the dermal papilla produces a tubelike epithelial structure that elongates and differentiates into a central part that will give rise to the rachis enclosed by the feather barbs and barbules (Lin, Foley, et al. 2013; Wu et al. 2021). To produce melanin‐pigmented feathers, melanin pigments are integrated into the keratin matrix of the feather barbs (Lin, Foley, et al. 2013; Lin, Wideliz, et al. 2013). To do this, melanocyte progenitors first migrate from the dermal papilla to the epithelial cylinder above the papilla ectoderm, where the melanocytes differentiate. Finally, the melanocytes mature and synthesise melanin, which is then transferred to keratinocytes and incorporated into keratin barbs (Lin, Foley, et al. 2013; Lin, Wideliz, et al. 2013). A non‐pigmented, white feather could therefore be produced by inhibiting any of these steps through a complex mechanism regulated temporally and spatially by paracrine signals from surrounding fibroblasts and keratinocytes (Lin, Foley, et al. 2013).

Among the regulators of melanin pigmentation, the agouti signalling protein (ASIP) and *POMC*‐derived melanocortin hormones (alpha‐MSH and ACTH) control melanin production by binding to the melanocortin 1 receptor, MC1R. In the case of a loss‐of‐function mutation in the proopiomelanocortin (*POMC*) gene, humans become deficient in melanocortin peptides, leading to the display of red hair and suffering from obesity and adrenal insufficiency (Krude et al. 1998).

In birds, the binding of melanocortin to MC1R induces the synthesis of eu‐ and pheomelanin pigments. Whereas the binding of its antagonist, Agouti (ASIP), results in either the absence of pigment deposition and thus partial or fully white feathers (Lin, Foley, et al. 2013; Roulin and Ducrest 2013), or the production of pheomelanin to the detriment of eumelanin (e.g., in the striped pattern of embryonic quail ( *Coturnix japonica* ) (Haupaix and Manceau 2020; Inaba and Chuong 2020)).

Single nucleotide polymorphisms (SNPs) in the *MC1R* gene are commonly associated with discrete colour variation in various bird species (Roulin and Ducrest 2013). In the European barn owl ( *Tyto alba* ), however, the valine to isoleucine transition at position 126 (*V126I*) on the *MC1R* gene is associated with a continuous variation of the ventral colouration from white to rufous, see fig 1 of San‐Jose et al. (2015). While individuals carrying both valine alleles (genotype referred to as *MC1R*_*VV*_) are whiter ventrally, the presence of one isoleucine is associated with darker rufous colouration (genotypes *MC1R*_*VI*_ and *MC1R*_*II*_). The fact that in the barn owl pheomelanic colouration varies continuously from white to rufous suggests that this trait is polygenic (San‐Jose and Roulin 2017). In addition, the white allele (*MC1R*_*VV*_) is associated with a greater colour variation (from white to slightly rufous) than the rufous alleles (*MC1R*_*VI*_ and *MC1R*_*II*_, encoding for a dark rufous colouration). The rufous allele therefore appears to hide (in part or entirely) the effect of other genes also controlling colouration (epistasis effect of *MC1R*) (San‐Jose, Ducret, Ducrest, Simon and Roulin 2017). Moreover, the *MC1R V126I* polymorphism explains around 30% of the variation in pheomelanin‐based colouration in the barn owl, and recently, two other genomic regions linked to pheomelanism were discovered, which explain together with *MC1R* around 80% of pheomelanin variation (Cumer et al. 2024). One region is located on the Z sex chromosome, and the other corresponds to *MATN2*, an autosomal gene.

Interestingly, the variation in pheomelanin colouration is associated with other phenotypic traits. Darker barn owls feed more upon common voles ( *Microtus arvalis* ) and whiter individuals on wood mice (*Apodemus* spp.) (Roulin 2004). In addition, darker individuals cope better with intense flight (Charter et al. 2015; Roulin et al. 2013) and settle further away from the birth site to reproduce 1 year later (natal dispersal) (Roulin 2013; van den Brink, Dreiss, and Roulin 2012). In some cases, darker pheomelanic males had higher reproductive success, a higher feeding rate (Roulin et al. 2001) and a lower probability of recruitment as a young breeder (Roulin and Altwegg 2007) demonstrating that colouration is not selectively neutral (Antoniazza et al. 2014). Darker nestlings are less aggressive against ornithologists who handle them in their hands and they are more cooperative by feeding their siblings (Roulin, Da Silva, and Ruppli 2012; Roulin et al. 2016) and express higher levels of 5a‐reductase (SRD5a2), an enzyme that metabolises testosterone into dihydrotestosterone (Beziers et al. 2017). The degree of pheomelanin‐based colouration is also negatively associated with oxidative stress (Roulin et al. 2011). Darker nestling females expressed higher levels of oestrogen receptor alpha (Beziers et al. 2017) and had larger ovaries (Roulin 2009). The basis of these relationships is still unknown, although pleiotropy or linkage disequilibrium might be driving these associations (Ducrest, Keller, and Roulin 2008; San‐Jose and Roulin 2018). A potential candidate is the melanocortin system, since melanocortin hormones regulate melanin synthesis and other processes, such as stress and immune responses, energy homoeostasis, steroidogenesis and exocrine functions (Ducrest, Keller, and Roulin 2008).

In the barn owl, our understanding of the effect of *MC1R* genotypes on feather pigmentation is limited to the expression of a set of well‐known melanin‐related genes detected using RT‐qPCR (Beziers et al. 2017; San‐Jose, Ducrest, et al. 2017). We found that rufous *MC1R* allele was associated not only with higher expression of genes downstream of the melanin pathway (*DCT*, *KIT*, *OCA2*, *SLC45A2*, *TYR* and *TYRP1*), but also with lower expression of *ASIP* and higher expression of *MC1R* itself. In addition, expression of *PCSK2*, the convertase that cleaves ACTH to produce the agonist of MC1R alpha‐MSH, was associated with the pheomelanin content in feathers of nestlings carrying the white *MC1R* allele but not the rufous *MC1R* allele. This suggests that MC1R regulates not only the expression of genes related to melanogenesis and melanocortin, but also the relationship between colouration and expression of genes upstream of the *MC1R* genes (San‐Jose, Ducrest, et al. 2017). To further examine which genes and how their expression is affected by *MC1R* genotypes during feather development, we performed RNA‐Seq analysis of growing feathers collected in pre‐fledgling males of *MC1R*_*VV*_ and *MC1R*_*VI*_ genotypes encoding for a whitish vs. rufous colouration. The RNA‐Seq method is adapted for discovering genes involved in melanogenesis (Liu et al. 2024; Wang et al. 2021, 2022; Zhou et al. 2022).

## 2 Materials and Methods

### 2.1 Tissue Sampling and Assessment of Melanin Pigments

The study was carried out in a population of wild barn owls in western Switzerland. For RNA‐Seq analysis, we collected blood and two to three developing chest feathers from 37 male nestlings (18 *MC1R*_*VI*_ and 19 *MC1R*_*VV*_) born in 2015 (the *MC1R*_*II*_ genotype could not be considered due to its low frequency (3.6%) in the Swiss population (San‐Jose et al. 2015)). We validated the RNA‐Seq results by using RT‐qPCR in an independent sample of 70 owls born in 2016 (27 males:23 *MC1R*_*VV*_ and 4 *MC1R*_*VI*_ and 43 females:29 *MC1R*_*VV*_ and 14 *MC1R*_*VI*_). For both experiments, we collected growing feathers from nestlings at a similar stage of development (mean age ± SD: 36 days ± 2.2 for the RNA‐Seq analysis and 38 days ± 3.5 for the RT‐qPCR analysis). At this age, the feathers are not yet fully grown, having developed the apical part of the feather but being in the process of developing most of the feather vane. Growing feathers could be sampled when the typical black spots on the apical part of the feather were already developed and thus when only the development of the white to rufous pheomelanin colouration is underway (most affected by *MC1R*, San‐Jose et al. 2015). Nevertheless, a picture of the basal part of each feather was taken at the time of RNA extraction, and feathers still developing a black spot were avoided for RNA‐Seq library preparation. Upon collection, feathers and blood were immediately frozen in dry ice and stored at −80°C until molecular analyses. For each individual, DNA extracted from blood samples was used for PCR‐based sexing and *MC1R* genotyping using previously described methods (Py et al. 2006; San‐Jose et al. 2015).

### 2.2 RNA‐Seq Library Preparation and Sequencing

Total RNAs were extracted using RNAeasy mini kit (Qiagen, Hombrechtikon, Switzerland) as described in (San‐Jose, Ducrest, et al. 2017) from at least one chest feather per individual. Each feather was extracted separately. RNA quality was measured using a fragment analyser (Advanced analytical, Labgene, Châtel‐St‐Denis, Switzerland) and samples with RQN values above 8.5 were used for library preparation (Table S1). Libraries were prepared using the Kapa stranded mRNA‐Seq Library Preparation kit (Roche, Basel, Switzerland). Briefly, mRNA was poly‐A selected from total RNA (500 ng) using poly‐T oligo‐attached magnetic beads. The Poly‐A RNA was eluted from the magnetic beads, fragmented into 300–400 bp and primed with random hexamers. The first strand of cDNA was synthesised using Kapa Script, then converted to blunt‐end fragments with A‐bases, followed by the synthesis of the second strand. A 350 nM Illumina PentAdapters (Illumina, Zurich, Switzerland) containing a T‐base overhang were ligated to the A‐tailed cDNA fragments. The ligated fragments were PCR‐amplified (12 cycles) and purified by Agencourt AMPure XP bead (Beckman Coulter Intl SA, Nyon, Switzerland). The length distribution of the libraries obtained was checked using a fragment analyser prior to sequencing.

Paired‐end reads of 100 bp were sequenced using a Hiseq 2500 at the University of Lausanne Genomic Facilities (GTF, Genopode, Lausanne, Switzerland). Approximately 74.4 ± 10 million raw reads were obtained per library (Table S2). The quality of the raw reads was assessed using FastQC v0.11.5 (Andrews 2010). The mean GC content was 51% ± 1 (mean ± standard deviation) for forward reads and 54% ± 1 for reverse reads. Reads were all of high quality, with an average of 91.99% ± 1.16 of bases above or equal to Q30. The adapters were trimmed with the Illuminaclip option of Trimmomatic 0.36 (Bolger, Lohse, and Usadel 2014). The raw reads were submitted in BioProject: PRJNA1119676 at NCBI, sample accession numbers from SAMN41661779 to SAMN41661811.

### 2.3 Read‐Mapping to the Reference Genome

Trimmed reads were mapped to the European barn owl ( *Tyto alba* ) genome (assembly T. alba_DEE_v4.0, GCA_018691265.1 (Machado et al. 2022)) using STAR 2.7.8a (Dobin and Gingeras 2015) with 2‐pass mode and polyA clipping and coordinate sorting of BAM files. On average, 86.6% ± 1.2% of the reads per library were mapped to the genome with an average of 57.3% ± 2.6 of the reads uniquely mapped (Table S2). A total of 18,889 genes were counted using HTSeq 0.11.2 (Anders, Pyl, and Huber 2015) with union mode. We also mapped the reads to the barn owl transcriptome using Kallisto v0.45.1 and R v4.3.1 (Bray et al. 2016a, 2016b). The Kallisto index was constructed with the reference GCF_018691265.1_T. alba_DEE_v4.0_rna and with kmer length of 31 (Machado et al. 2022). The pseudoalignment rate was 82.02% ± 1.45 with 43.02% ± 1.80 of reads uniquely mapped.

### 2.4 Differential Expression Analyses

DESeq2 v1.30.1 (Love, Huber, and Anders 2014) in R 4.1.2 was used to test differential gene expression separately for counts obtained with STAR‐HTSeq and with Kallisto. Preliminary analyses with Pheatmap and PCA in DESeq2 identified four outlier libraries (L4, L8, L14, L29) that showed a distinct profile of gene expression compared to the rest of the libraries and we removed them for further analysis. Examination of photographs taken of the basal part of the feather prior to RNA extraction suggests that the feather bases were missing for these libraries, probably broken during flash freezing or stuck to the wall of the tube prior to extraction. We set a False Discovery Rate (FDR) of 0.05 to identify differentially expressed (DE) genes between *MC1R* genotypes. The results of the differential expression analysis can be found for STAR‐ HTSeq and Kallisto in Tables S4 and S5, respectively. The 33 nestlings considered in the RNA‐Seq analysis came from 23 broods with each time one individual and 5 broods with each time two siblings (Table S1). Because we do not have enough pairs of siblings, we could not take brood identity into account in the DESeq2 models. Nevertheless, to evaluate the potential effect of brood identity on detecting differential gene expression, we randomly selected one chick from the 5 broods with more than one individual analysed. Then, we performed the Kallisto DESeq2 analysis three times with different chick combinations. We then compared the results of the three trials based on 28 individuals with the results obtained in the first analysis considering all 33 nestlings, that is, which genes were differentially expressed between *MC1R* genotypes (Tables S6 and S7). Apart from *TBXA2R*, *RAB3C* and *VSTM2L* which were only found in the analysis with 33 nestlings, all the other genes were found again in at least one of the three trials with 28 nestlings. This means that the results obtained without taking account the effect of brood identity remain valid.

### 2.5 Gene Set Enrichment Analysis

Gene ontology (GO) analyses were conducted with the R package topGO (version 2.56.0), (Alexa and Rahnenfuhrer 2024). The chicken was used as the reference species (org.Gg.eg.db v3.19.1). Out of 16,890 Kallisto genes, 10,966 orthologs were retrieved from chicken and used as background for the analysis. Among the 21 Kallisto differentially expressed genes, which are common to STAR‐HTSeq: *LOC116960911*: *killer cell lectin‐like receptor subfamily B member 1B allele C* (*KLRB1*) and *LOC116960306: flocculation protein* (*FLO11‐like*) and *RAB3C* were not found in the chicken orthologs. In addition, for the Kallisto gene *SLC28A2*, there was no chicken ortholog. We tested enrichment by scoring GO terms using the elim and weight01 algorithms and using *p*‐values of weighted Fisher results with scores ≤ 0.01 (Alexa, Rahnenfuhrer, and Lengauer 2006). The elim and weight01 functions take into account the intercorrelated hierarchy between terms, thereby reducing the false positive rate (Alexa, Rahnenfuhrer, and Lengauer 2006).

### 2.6 Weighted Gene Co‐Expression Network Analysis

To support the RNA‐Seq results, we ran a weighted gene co‐expression network analysis (WGCNA) on all the genes counted by Kallisto DEseq2. The correlation network analysis was performed with WGCNA v1.70‐3 in R excluding the RNA‐Seq 26 library which was an outlier in the quality control of library homogeneity on normalised DEseq2 genes, as recommended by Langfelder and Horvath (2008). We constructed an unsigned network, based on Pearson and a power of 7, which is also the recommended power for more than 30 samples (Langfelder and Horvath 2008). A minimum module size of 25 and merge cut height of 0.15 were used to construct the network. After computing the dissimilarity between genes, the hierarchical clustering of genes, dividing their clusters into modules and finally merging similar modules, we obtained 27 different modules. The module eigengenes that could be considered as the gene profiles of the modules were then tested for their association with *MC1R* genotypes. To find the genes most associated with *MC1R* in each module, we looked for genes that were strongly connected within the module and that correlated most strongly with *MC1R* genotypes represented in the WGCNA by high gene module membership and high gene significance value, respectively. For gene significance, we determined the *Cohen's d* effect size coefficient for each gene in the module, and for gene module membership, we estimated it using Spearman's correlation coefficients. Finally, the ‘blue sky’ module was visualised using Cytoscape v3.9.1 (Shannon et al. 2003). The most significant gene and module membership were selected for an absolute value greater than 0.9 quantile.

### 2.7 Validation of the New Genes With RT‐qPCR

An independent experiment with 70 male and female nestling barn owls was designed to validate some of the genes differentially expressed between the *MC1R*_*VV*_ and the *MC1R*_*VI*_ genotypes identified by RNA‐Seq. The expression levels of the following genes were measured using RT‐qPCR: *CALB1*, *DDC*, *EEF1A*, *FNDC9L*, *GAPDH*, *GPR143*, *MC1R*, *MFSD12*, *MLANA*, *MITF*, *PMEL17*, *TYR*, *SLC45A2*, *RAB38*, *RPL13*, *SLC6A4*, *SYNGR3*, *TBP*, *TBXA2R*, *VAT1L* and *VIP*. During RNA extraction, genomic DNA was removed in two steps: first using the g‐DNA eliminator column of the RNeasy mini plus kit (Qiagen, Hombrechtikon, Switzerland) using RLT (RLT plus buffer reduced the amount of extracted feather RNA by 90%), and then using the Qiagen's DNAse I treatment on the column. Next, we reverse‐transcribed 1 μg total RNA in 20 μL using Vilo Superscript III Master mix (Life technologies, Thermo‐Fisher Scientific, Switzerland) by incubating for 10 min at 25°C, 1 h at 42°C, and stopped the reaction for 5 min at 85°C. Due to the low expression of *MC1R*, 13 μL of cDNAs were preamplified with all the primers of the different genes for the qPCRs for 14 cycles (Life technologies, Thermo‐Fisher Scientific, Switzerland) and diluted 10 fold in 10 mM Tris–HCl pH 8.0 and 0.1 mM EDTA and frozen at −20°C until the qPCR experiments. Preamplification did not affect gene expression as assessed by San‐Jose, Ducrest, et al. (2017). For qPCR, we set up qPCR conditions with different concentrations of primers and probes with different concentrations of templates (plasmids or PCR purified products) to achieve PCR efficiency between 95% and 105% (Table S3 and (Beziers et al. 2019; San‐Jose, Ducrest, et al. 2017)). The qPCRs were run in triplicate on QuantStudio Q6 (Thermo‐Fisher Scientific, Switzerland) using 1× qPCR Mastermix plus low Rox (Eurogentec SA, Liège, Belgium) in a final volume of 10 μL with 1 μL of pre‐amplified cDNA. When Ct values for triplicates differed by more than 0.3 Ct, the qPCR was repeated. To control for variation between plates, three pools of different pre‐amplified cDNAs were introduced into each plate. qBasePLUS 1.3 software (Biogazelle, Zwijnaarde, Belgium) was used to calculate target gene expression relative to the following reference genes: *Elongation factor 1A* (*EEF1A*) and *TATA‐Box‐binding protein* (*TBP*). These two genes had the lowest GeNorm M value. The GeNorm M values for *EEF1A* and *TBP* were 0.23 and 0.245, respectively, and the GeNorm V value was 0.0825. Mean relative quantities (RQ) values were used in subsequent statistical analyses. To determine whether *MC1R* genotypes affect gene expression levels, we ran a linear mixed model (lme function, ‘nlme’ package, version 3.1‐166, https://svn.r‐project.org/R‐packages/trunk/nlme/) to test for differences between *MC1R* genotypes on each of the qPCR candidate genes. The models included *MC1R* genotype and sex as predictors and ‘brood origin’ as a random factor. In the results, the interactions between sex and *MC1R* genotypes were not considered as they were never significant (*t*‐value range for all genes −1.94 to 1.82, *p*‐values > 0.059, *df* = 43). The *p*‐values were corrected for multiple testing (FDR). qPCR data were log +1 transformed to normalise the expression levels using R 4.1.2 (R Core Team, Vienna, Austria).

## 3 Results

### 3.1 Identification of Differentially Expressed Genes by RNA‐Seq and Validation by RT‐qPCR

**FIGURE 1:**
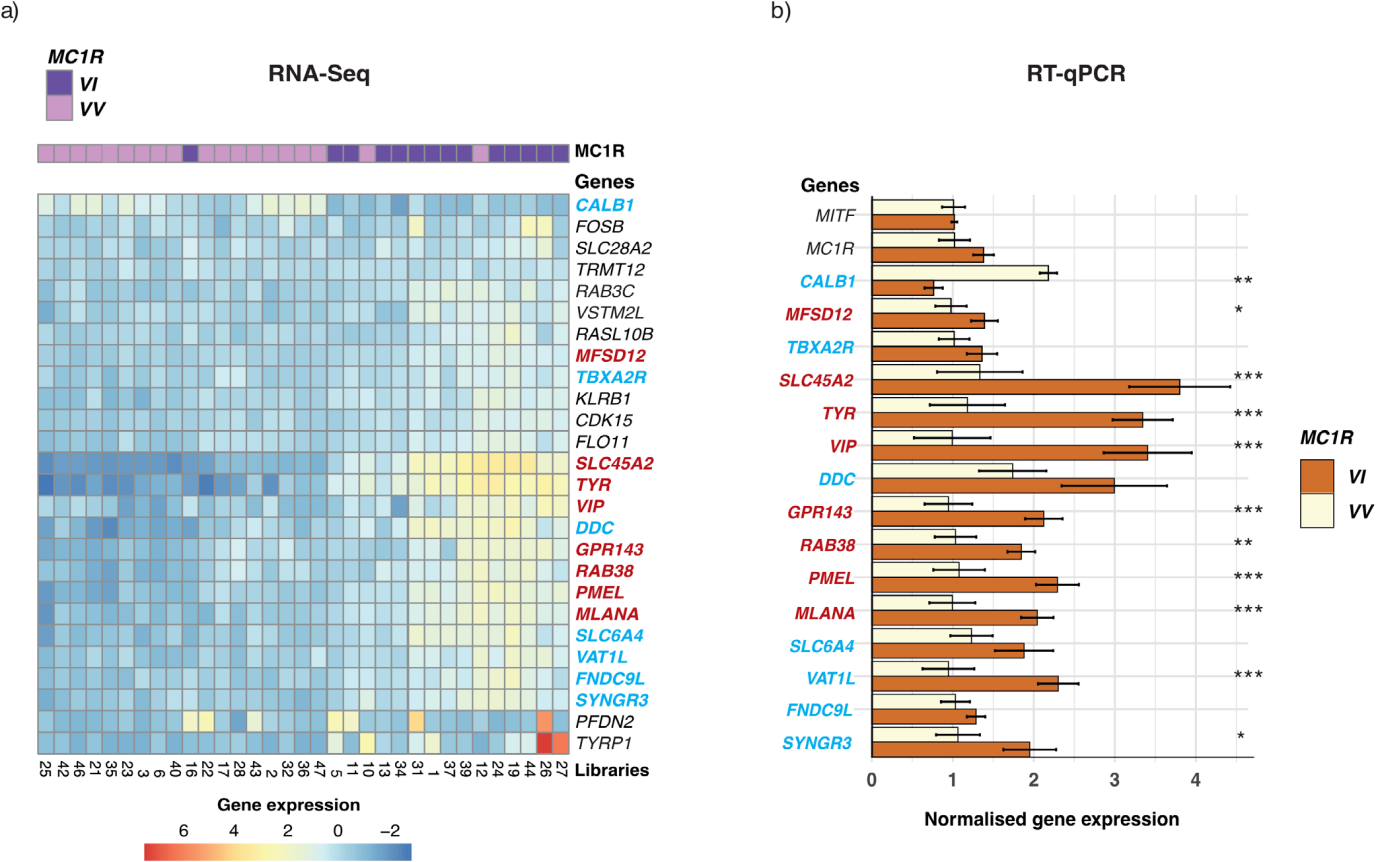
Differences in feather gene expression between *MC1R* genotypes. (a) Normalised gene expression of differentially expressed genes detected with Kallisto‐DESeq2 for the different libraries. (b) Normalised gene expression of some genes verified by RT‐qPCR in 70 other *MC1R*_*VV*_ individuals (*n* = 56) and *MC1R*_*VI*_ (*n* = 18). In red, the melanin‐related genes, in blue, the non‐melanic genes present in both experiments. Mean values (± SE) of normalised gene expression are reported; asterisks indicate significant differences between *MC1R* genotypes (adjusted *p*‐values: *p < 0.05, **p < 0.01, ***p < 0.001).

We detected 23 and 25 differentially expressed genes between white *MC1R*_*VV*_ and rufous *MC1R*_*VI*_ nestlings using STAR‐HTSEQ2 and Kallisto‐derived counts, respectively. Of these, 21 were found with both counting methods and include 9 well‐known melanogenic genes: *G Protein‐coupled receptor 143* (*GPR143/OA1*) (McKay 2019), *Major Facilitator Superfamily Domain Containing 12* (*MFSD12*) (Crawford et al. 2017), *Melanoma antigen recognised by T cells 1* (*MLANA/MART1*) (Aydin et al. 2012), melanocyte protein (*PMEL/gp100*) (Bissig, Rochin, and van Niel 2016), *RAB38, Member RAS Oncogene Family* (*RAB38*) (Loftus et al. 2002), *Solute carrier family 45 member 2* (*SLC45A2*), *Tyrosinase* (*TYR*), *Tyrosinase‐related protein 1* (*TYRP1*) (Ito and Wakamatsu 2011) and LOC104368820/LOC122153719*: Vasoactive Intestinal Peptide* (*VIP*) (Yuan et al. 2016). All these genes were up‐regulated in the growing feathers of rufous *MC1R*_*VI*_ nestlings compared with *MC1R*_*VV*_ nestlings (Figure 1a and Table S6).

The non‐melanin‐related genes are *Calbindin 1* (*CALB1*), *Aromatic Amino Acid Dopa Decarboxylase* (*AADC*/*DDC*), LOC116960306: *flocculation protein‐like* (*FLO11*), LOC104357634: *Fibronectin Type III Domain Containing 9‐like* (*FNDC9l*), *LOC116960911*: *killer cell lectin‐like receptor subfamily B member 1B allele C* (*KLRB1*), *Ras‐Related Protein Rab‐3C* (*RAB3C*), *RAS Like Family 10 Member B* (*RASL10B*), *Solute carrier family 6 member 4* (*SLC6A4/SERT*), *Synaptogyrin 3* (*SYNGR3*), *Thromboxane A2 Receptor* (*TBXA2R*), *Vesicle Amine Transport 1‐like* (*VAT1L*) and *V‐Set And Transmembrane Domain Containing 2 Like* (*VSTM2L*). These genes were up‐regulated in the growing feathers of *MC1R*_*VI*_ nestlings, except for *CALB1* (up‐regulated in the growing feathers of white *MC1R*_*VV*_ nestlings). The other two up‐regulated genes found in *MC1R*_*VI*_ with STAR‐HTSEQ2 DESeq2 are the *Serine/Threonine Kinase 32A* (*STK32A*) which is down‐regulated in albino Russian sturgeon (Gong et al. 2019) and *Serpin Family E Member 3* (*SERPINE3*) which regulates the melanin cascade in *O. furnacalis* pest larvae (Chu et al. 2015). In Kallisto DESeq2, the other four genes up‐regulated in *MC1R*_*VI*_ are the *Cyclin Dependent Kinase* (*CDK15*), the LOC122152587: *prefoldin subunit 2‐like* (*PFDN2‐L*), the *Solute Carrier Family 28 Member 2* (*SLC28A2*), a nucleoside transporter and *FOSB*, a transcription factor part of the AP1 complex that regulates melanosome maturation and melanin synthesis (Campagne et al. 2018).

Using growing feathers from a further 70 male and female nestlings, we tested whether *CALB1*, *DDC*, *FNDC9L*, *GPR143*, *MFSD12*, *MLANA*, *PMEL*, *RAB38*, *SLC45A2*, *SLC6A4*, *SYNGR3*, *TBXA2R*, *TYR*, *VAT1L* and *VIP* genes differentially expressed by RNA‐Seq were also differentially expressed by RT‐qPCR. The results show the same expression trend as that obtained with the RNA‐Seq data, with significant differences for *CALB1*, *GPR143*, *MFSD12*, *MLANA*, *PMEL, RAB38*, *SLC45A2*, *TYR*, *VAT1L* and *VIP* (*p*‐values are FDR corrected from linear mixed models, Figure 1b). All these genes are more expressed in rufous *MC1R*_*VI*_ than *MC1R*_*VV*_ individuals, except for *CALB1*, which is more expressed in whiter individuals, as also shown in the RNA‐Seq study.

### 3.2 Gene Set Enrichment Analysis of the Differentially Expressed Genes

The differentially expressed genes found with Kallisto DEseq were significantly enriched for the biological process categories of gene ontology related to melanin pigmentation (melanosome organisation, melanin biosynthesis processes, Table 1). For non‐melanin‐related function, *VIP* contributes to six different GO terms: positive regulation of epinephrine secretion, sensory perception of pain, penile erection, learning and memory and negative regulation of smooth muscle cell proliferation and, with *TBXA2R*, cAMP levels. *CALB1* is linked to synapse potentiation and presynaptic calcium concentration, *DDC* to the biosynthesis of the neurotransmitters catecholamines and serotonin and *SLC6A4* to serotonin.

**TABLE 1:** Biological processes enriched in differentially expressed genes between MC1R genotypes sorted by *p* values.

GO_ID	Term	Annotated	Significant	Expected	*p*	Genes
GO:0032438	Melanosome organisation	4	3	0.01	1.50E‐08	*GPR143, TYRP1, PMEL*
GO:0032812	Positive regulation of epinephrine secretion	1	1	0	0.0017	*VIP*
GO:0048662	Negative regulation of smooth muscle cell proliferation	1	1	0	0.0017	*VIP*
GO:0051610	Serotonin uptake	1	1	0	0.0017	*SLC6A4*
GO:0051930	Regulation of sensory perception of pain	1	1	0	0.0017	*VIP*
GO:0060406	Positive regulation of penile erection	1	1	0	0.0017	*VIP*
GO:0045777	Positive regulation of blood pressure	2	1	0	0.0034	*TBXA2R*
GO:0007189	Adenylate cyclase‐activating G protein‐coupled receptor signalling pathway	58	2	0.1	0.0042	*VIP, TBXA2R*
GO:0042438	Melanin biosynthetic process	3	1	0.01	0.0051	*TYR*
GO:0050848	Regulation of calcium‐mediated signalling	3	1	0.01	0.0051	*GPR143*
GO:0099509	Regulation of presynaptic cytosolic calcium ion concentration	3	1	0.01	0.0051	*CALB1*
GO:1900271	Regulation of long‐term synaptic potentiation	3	1	0.01	0.0051	*CALB1*
GO:0042427	Serotonin biosynthetic process	4	1	0.01	0.0068	*DDC*
GO:0006584	Catecholamine metabolic process	5	1	0.01	0.0084	*DDC*
GO:0007611	Learning or memory	5	1	0.01	0.0084	*VIP*
GO:0090385	Phagosome‐lysosome fusion	5	1	0.01	0.0084	*RAB38*

*Note:* Annotated: number of genes present in the Kallisto‐DESeq2 analysis; Significant: number of genes differentially expressed; Expected: The number of differentially expressed genes that would be expected by chance if differential expression were randomly distributed among all annotated genes.

### 3.3 Weighted Gene Co‐Expression Network Analysis

**FIGURE 2:**
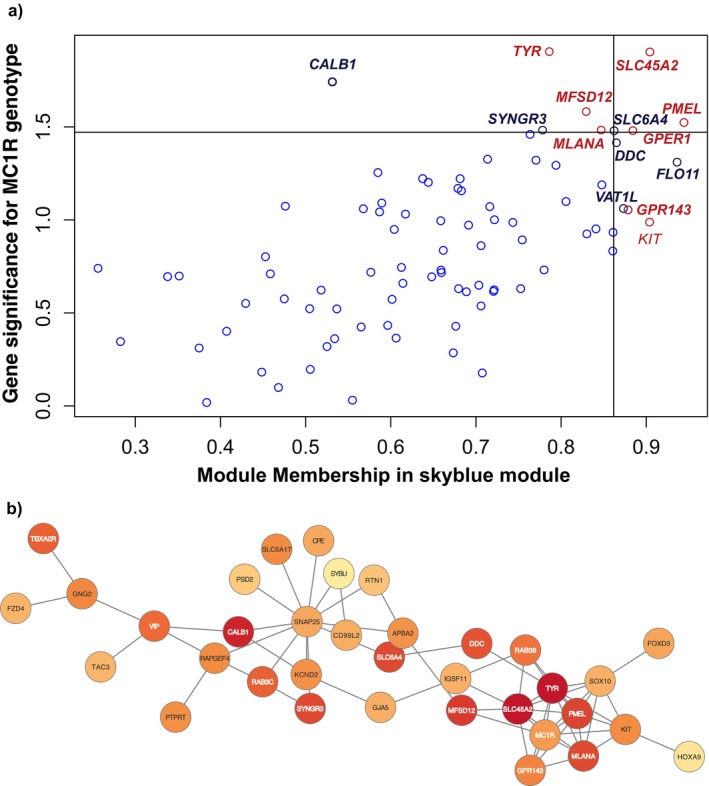
Weighted gene co‐expression network analysis (WGCNA) of genes of the Kallisto‐DESeq2 method. (a) Relationship between gene significance, which measures the association between genes and *MC1R* genotypes with Cohen's d coefficient of effect size, and module membership: Measured by Pearson correlation that is the strength of association of genes with the sky‐blue module (absolute values are presented). The most influential genes whose module membership and/or gene significance is greater than the 0.9 quantile are labelled, with genes involved in the melanogenic pathway highlighted in red italic and underlined in bold when differentially expressed. Differentially expressed genes not involved in the melanogenic pathways are highlighted in bold black. (b) Visualisation of the sky‐blue module gene relationship with Cytoscape. The differentially expressed genes of the RNA‐Seq data are written in white. The colour of the circle is proportional to the absolute gene significance, the darker the more significant.

To confirm the relationship between melanic and non‐melanic genes detected in differentially expressed genes, we performed a weighted gene co‐expression network analysis (WGCNA) on all the genes counted by Kallisto DESeq2 (Figure 2 and Figure S1). Two modules were associated with *MC1R* genotypes: light sky‐blue (*r* = −0.56, *p* = 0.00097) and dark olive green (*r* = −0.42, *p* = 0.015). The sky‐blue module contains 85 genes, including 19 out of the 21 differentially expressed genes found in both Kallisto‐ and STAR‐HTSEQ2‐derived counts (Table 2). The genes in the module are significantly associated with *MC1R* genotypes (Cohen's d effect size coefficient = −1.316, 95% CI: −2.106, −0.526, Student's‐*t*_30_ = 3.66, *p* = 0.001, Figure 2a). The following 12 differentially expressed genes (*CALB1*, *DDC*, *GPR143*, *LOC116960306*/*FLO11*, *MFSD12*, *MLANA*, *PMEL*, *SLC45A2*, *SLC6A4*, *SYNGR3*, *TYR* and *VAT1L*) were among the most relevant genes for the module, with module membership and/or gene significance above the 0.9 quantile (Figure 2a). The other genes present in the module and important for melanogenesis were *MC1R* and *SOX10* (Table 2). Indeed, the sky‐blue module is enriched in genes with the following main biological functions: pigment biosynthetic process, cell–cell signalling, regulation of biological quality, developmental pigmentation, system process, organic hydroxy compound biosynthetic process, adenylate cyclase‐activating G protein‐coupled receptor signalling pathway and regulation of nervous system development, some of the GO terms of the biological processes similar to those found with genes differentially expressed by RNA‐Seq.

**TABLE 2:** *MC1R* genotypes derived gene significance and module membership of the WGCNA sky‐blue module containing the differentially expressed genes found with the Kallisto‐DESeq2 method.

Genes	Module membership	Gene significance
*ACOX2*	−0.679	1.169
*AMIGO3*	0.577	−0.719
*APBA2*	0.83	−0.925
*ATP6V1C2*	0.841	−0.952
*CALB1*	−0.532	1.75
*CD99L2*	0.703	−0.649
*CDH18*	−0.352	0.698
*CDK15*	0.806	−1.099
***CPE***	0.648	−0.694
*CUNH15orf62*	−0.706	0.538
*DDC*	0.873	−1.416
*EMILIN3*	0.589	−1.091
***FOXD3***	0.78	−0.731
***FZD4***	0.43	−0.551
*GDPD4*	0.707	−0.177
*GFOD2*	−0.676	0.428
***GJA5***	0.68	−0.63
*GNG2*	0.617	−1.031
***GPER1***	0.862	−1.48
***GPR143***	0.873	−1.061
*HOXA9*	0.468	−0.099
*IFITM10*	0.659	−0.717
***IGSF11***	0.721	−0.616
*IREB2*	−0.602	0.573
*KCND2*	0.861	−0.933
***KIT***	0.904	−0.988
*KLHL32*	0.555	−0.031
*LOC104357024/SSX2IP*	−0.256	0.74
*LOC104357634/FNDC9l*	0.764	−1.46
*LOC104366696/TRPV2*	0.706	−0.861
*LOC104368820/VIP*	0.585	−1.254
*LOC104369395/TBC1D24L*	−0.505	0.523
*LOC116958914*	0.659	−0.73
*LOC116960293*	0.689	−0.614
*LOC116960306/FLO11*	0.936	−1.311
*LOC116960911/KLRB1*	0.794	−1.293
*LOC116961354/HEXDCL*	0.518	−0.623
*LOC116962307*	0.375	−0.311
*LOC116962430*	0.673	−0.285
*LOC116963363*	−0.613	0.745
*LOC116964843*	0.453	−0.802
*LOC116965663*	0.525	−0.319
*LOC122152844/AARSD1L*	−0.459	0.71
*LOC122153475*	−0.449	0.182
*LOC122153970*	−0.283	0.346
*LOC122154150*	0.614	−0.659
*LOC122154315*	−0.537	0.522
***MC1R***	0.861	−0.833
*MESD*	−0.604	0.949
***MFSD12***	0.837	−1.587
***MLANA***	0.854	−1.485
*MTMR2*	0.662	−0.836
*MYOM1*	−0.407	0.401
*NEURL1*	0.587	−1.043
*PAPSS1*	0.534	−0.361
*PLPPR1*	0.596	−0.433
***PMEL***	0.944	−1.524
*PMS1*	−0.659	0.995
*PSD2*	0.606	−0.365
*PTPRT*	0.691	−0.972
*QPCT*	0.754	−0.893
***RAB38***	0.848	−1.188
*RAB3C*	0.771	−1.321
*RAPGEF4*	0.743	−0.986
*RASL10B*	0.644	−1.202
*RASSF2*	0.568	−1.06
*RETSAT*	0.683	−1.157
*RPS6KA2*	0.753	−0.63
*RTN1*	0.565	−0.425
*SEMA6D*	−0.681	1.222
***SLC45A2***	0.904	−1.903
*SLC6A17*	0.722	−1.001
*SLC6A4*	0.893	−1.482
*SNAP25*	−0.338	0.695
*SOSTDC1*	0.505	−0.196
***SOX10***	0.721	−0.624
*STAC2*	−0.476	1.074
*SYBU*	0.384	−0.018
*SYNGR3*	0.784	−1.485
*TAC3*	−0.475	0.575
*TBXA2R*	0.714	−1.326
*TSPAN10*	0.716	−1.071
***TYR***	0.792	−1.917
*VAT1L*	0.879	−1.054
*VSTM2L*	0.637	−1.222

*Note:* Differentially expressed genes are highlighted in green and melanic genes are written in bold. Gene trait significance corresponds to the Cohen's *D* effect size coefficient, the Module Membership was calculated with the Spearman Rho correlation coefficient.

A second significant WGCNA module (dark‐olive green, Cohens' *d*: −0.925, 95% CI −1.679, −0.171, Student's‐*t*_30_ = 2.57, *p* = 0.0154) containing 32 genes is associated with the *MC1R* genotype but was not highlighted by the differential expression analysis (Table S8). These genes are involved in carbon dioxide transport, myeloid cell development, nitric oxide transport, hydrogen peroxide catabolic process, localisation, cellular oxidant detoxification and blood coagulation, and their relationships with *MC1R* genotypes need to be further investigated.

## 4 Discussion

Previously, we found that *MC1R* genotypes influence the expression of melanin and melanocortin‐related genes, as well as their co‐expression in barn owl feathers (San‐Jose, Ducrest, et al. 2017). Here, RNA‐Seq and qPCR data not only confirmed that *MC1R*_*VI*_ rufous nestling barn owls express higher levels of melanin‐related genes such as *SLC45A2*, *TYR*, *TYRP1*, *PMEL* and *MLANA* (Beziers et al. 2019; San‐Jose, Ducrest, et al. 2017) but allowed us to discover other melanin‐related genes (*GPR143, MFSD12*, *RAB38* and *VIP*) whose expression is also up‐regulated in the feathers of rufous *MC1R*_*VI*_ individuals (Figure 1a,b). Furthermore, our RNA‐Seq study indicates that *MC1R*_*VI*_ rufous owls can also express higher levels of *DDC*, *FNDC9L*, *SLC6A4*, *SYNGR3, VAT1L* and lower levels of *CALB1*. A similar result was observed with an independent set of individuals by RT‐qPCR (Figure 1a,b) suggesting indirect links between these genes and melanogenesis.

Interestingly, these other differentially expressed genes (namely *DDC*, *SYNGR3*, *RAB3C*, *SLC6A4, VAT1L, VSTM2L* and *CALB1*) are involved in neurotransmitter pathways or synaptic influx (Brimblecombe et al. 2019; McInnes et al. 2018; Nagatsu 1991; Rossini et al. 2011; Schiavo and Stenbeck 1998; Xu, Wang, and Yao 2019; Yang et al. 2019). In addition, another gene also up‐regulated in *MC1R*_*VI*_ individuals, VIP, is a neuropeptide with multiple functions. In birds, VIP has been shown to have a wide distribution in the brain (Kuenzel 2003) and it plays a role in prolactin secretion, brooding and moulting (Kuenzel 2003; Sharp, Dawson, and Lea 1998). In addition, it is expressed in the pineal gland and may control melatonin production in chicken (Adamska et al. 2018). It has been involved in growth in Japanese quails (Bai et al. 2023; Bohler, Gilbert, and Cline 2021), in aggressive and grouping behaviour outside breeding season in sparrows (Goodson, Wilson, and Schrock 2012) and it stimulates the exocrine pancreas in ducks (Wang and Cui 2007). In other vertebrates, it has been associated with melanogenesis in *Xenopus laevis* cells, regulating POMC expression in skin melanophores (Kidane et al. 2007). In addition, VIP increases ACTH levels in neonate rats (Bodnar et al. 1997) and induces melanin synthesis via up‐regulation of CREB, MITF and Tyr in murine melanoma cell lines (B16F10; (Yuan et al. 2016)). Other differentially expressed genes: *DDC*, *SLC6A4*, *SYNGR3* and *CALB1*, are involved in the metabolism of the neurotransmitters dopamine, serotonin and melatonin (all known modulators of melanogenesis; (Slominski et al. 2004)). Ddc (or Aad) catalyses the decarboxylation of L‐3,4‐dihydroxyphenylalanine (L‐DOPA) to dopamine, L‐5‐hydroxytryptophan to serotonin (5‐HT) and L‐tryptophan to tryptamine (Bertoldi 2014). *DDC* was shown to be expressed in melanocytes and keratinocytes (Gillbro et al. 2004). Slc6a4, also known as 5‐HTT or SERT, is an integral membrane protein that terminates and recycles serotonin in presynaptic vesicles in a sodium‐dependent manner (Moller et al. 2019) and stores 5‐HT in some cells, such as platelets (Rudnick and Sandtner 2019), human melanoma and keratinocyte cell lines (Slominski et al. 2012). This gene was also differentially expressed in the hind legs of dispersing versus non‐dispersing common lizards (*Zootoca vivipara*, (San‐Jose et al. 2023)). Syngr3 is an integral membrane protein that regulates the dopamine transporter (DAT) at presynaptic terminals (Egana et al. 2009). Finally, Calb1, also known as Calbindin‐D28K, acts as a mobile Ca^2+^ buffer at nerve terminals and modulates dopamine release (Brimblecombe et al. 2019; Pan and Ryan 2012).

Expressions of *TBXA2R* and *FNDC9*‐l (up‐regulated in *MC1R*_*VI*_ rufous nestling feathers) have previously been detected in mammalian melanocytes, although their role in melanin synthesis is unclear. TBXA2R is a membrane GPCR receptor for thromboxane A2, expressed in hair follicles (Colombe, Michelet, and Bernard 2008), which may stimulate melanocyte growth and dendrite formation (Hu, McCormick, and Woodward 2001). Fibronectin type III domain‐containing protein 9‐like (FNDC9‐l) could be part of the extracellular matrix. Fibronectins are secreted by human melanocytes after UVB irradiation in hyperpigmented lesions (Bin et al. 2016) and fibronectins play a role in melanocyte migration and differentiation during embryogenesis (Takano et al. 2002). Our study additionally supports the role of these genes in pigmentation in birds.

The remaining differentially expressed genes—*VAT1L* and *VSTM2L*—are less well characterised, and to our knowledge, no direct or indirect link with melanin synthesis has been established previously. However, interestingly, these genes have been connected to neurotransmitters. Both are present at nerve endings; V‐set and transmembrane domain‐containing two like (VSTM2L, also known as C20orf102), is a secreted protein that modulates the activity of humanin in neural cells and is involved in neurodegenerative diseases and cancer (Rossini et al. 2011; Zhang et al. 2021). The synaptic vesicle membrane protein VAT‐1 homolog‐like (VAT1L) has been associated with schizophrenia (Chang et al. 2015) and VAT1, its paralog, is responsible for the storage and release of neurotransmitters in synapses (Levius and Linial 1993) and its activity in keratinocytes is calcium‐dependent (Koch et al. 2003). Interestingly, another study revealed that *VAT1L* is differentially expressed in mice hair follicle melanocytes (Infarinato et al. 2020). Rab3c is a small GTPases of the RAB3 family, involved in exocytosis. It also regulates neurotransmitter release into synaptic vesicles and Ca^2+^ entry (Schiavo and Stenbeck 1998; Schonn et al. 2010).

In general, most of the non‐melanic related genes that were differentially expressed in *MC1R*_*VI*_ rufous owl feathers are neurotransmitter‐related genes and, in particular, catecholamine and serotonin regulatory pathways (with the exception of *RASL10B*, *FLO‐11* and *KLRB1*, which are a small GTPase (Zou et al. 2006), a probable adhesion protein (Willaert 2018) and a stimulator of cytotoxic response of NK cells (Konduri et al. 2020), respectively). The presence of these genes in our experiment could be explained by the fact that we did not isolate melanocytes but used the embedded tips of one plucked feather per individual. The tips contain the follicular sheet, the epidermal collar, part of the dermal papilla and the pulp of the growing feather, so we could also have measured the expression of paracrine factors controlling melanin production and feather growth in the feather follicle. The pulp of growing feathers contains blood vessels (Wu et al. 2021), as well as cells such as keratinocytes, fibroblasts, Schwann cells, Merkel cells and melanocytes (Slominski et al. 2022). The nerve terminals form a ‘nerve ring’ around the feather follicles in chicken (Pays et al. 1997) and are connected to the follicular sheath (Duc, Barakat‐Walter, and Droz 1993; Weir and Lunam 2006). Recently, the sympathetic nervous system has been shown to regulate the proliferation of melanocyte stem cells in mouse hairs (Zhang et al. 2020). Interestingly, in non‐breeding crested ibis ( *Nipponia nippon* ), feather regeneration was also associated to the up‐regulation of neuron‐related genes (Sun et al. 2020). Therefore, it is likely that the expression of neurotransmitter‐related genes is likely in the embedded tips of plucked feathers. Although, this does not explain the higher expression of neurotransmitter‐related genes for individuals carrying the rufous *MC1R*_*VI*_ variant. Indeed, it has been observed that the concentration of nerve endings in contact with melanocytes increases after UV‐A irradiation (Kumakiri, Hashimoto, and Willis 1978), suggesting that melanin synthesis is associated with the expression of molecules responsible for nerve ending formation and hence neurotransmitter transporters in humans. In addition, it is also possible that follicles producing more melanin require greater coordination between follicular cells than follicles producing white feathers, since the melanoblasts must proliferate, migrate upwards, produce melanin and transfer their melanin to the surrounding keratinocytes. All these steps are controlled by paracrine signals, some of which remain unknown (Cui and Man 2023). In white barn owls, melanocytes are arrested in one of these steps, which remains to be determined. In contrast, in rufous *MC1R*_*VI*_ feathers, *MC1R* or another downstream melanin‐related gene would not only be a direct key player in melanin synthesis but also a regulator of *MC1R* upstream regulators. Indeed, we have previously shown that in barn owl feathers, the expression of certain melanocortin‐related genes depends on the *MC1R* genotype (San‐Jose, Ducrest, et al. 2017). The splice variant *ASIP‐AC* has reduced expression in *MC1R*_*VI*_ owls and *MC1R* expression itself increases in this genotype. This suggests a spatiotemporal positive feedback loop that supports eu‐ and pheomelanin synthesis in melanocytes during the growth of the rufous feather part. Similarly, Cui et al. (Cui and Man 2023) showed that non‐functional *MC1R* mutant mice have a lower number of melanoblast stem cells migrating into the skin after wounding compared to mice with wild‐type *MC1R*. This further suggests that the MC1R activity directly or indirectly controls melanocyte differentiation and maturation.

One potential regulator is the microphthalmia‐associated transcription factor (*MITF*) gene, which acts downstream of MC1R and is responsible for melanocyte survival, proliferation and melanin synthesis. This gene is already expressed early in the embryo in the neural crest cells that give rise to melanocytes (Cui and Man 2023). Interestingly, in humans, *SLC6A4*, *DDC*, *RAB3C*, *RASL10B*, *SYNGR3*, *TBXA2R*, *VAT1* all contain binding sites occupied by MITF (as shown in a ChIP‐seq experiment in human 501Mel cell line; (Strub et al. 2011)). This would suggest that these genes were differentially expressed in owls with distinct *MC1R* genotypes, as they would not only be expressed in melanocytes, but also activated by MC1R‐stimulated MITF (although occupancy of MITF binding is no guarantee of transcriptional activation (Goding and Arnheiter 2019) and other activators probably need to fire at the same time to start their gene transcription).

Correlation network analysis indicates the association between the sky‐blue module and *MC1R* genotypes is not centred on the *MC1R* or *MITF* genes, but rather on key melanogenic genes: *SLC45A2, PMEL, GPR143, MLANA, MFSD12* and *TYR*. *MITF* and *MC1R* are not among the differentially expressed genes, nor are they the most significant genes of the module (Table 2 and Figure 2a,b). On the basis of this result, we could also hypothesise that melanin synthesis and the genes involved in this synthesis are responsible for the differential expression of neurotransmitter‐related genes in rufous *MC1R*_*VI*_ feathers. One molecule that could potentially mediate melanin synthesis and neurotransmitter‐related gene expression is L‐DOPA (L‐dihydroxyphenylalanine), which is produced during melanin synthesis (Slominski, Zmijewski, and Pawelek 2012). During melanin synthesis, Tyr oxidises L‐tyrosine to dopaquinone, which is highly reactive. In the presence of sulfhydryl compounds, the reaction continues, giving rise to pheomelanin molecules. In the absence of thiol groups, dopaquinone undergoes intramolecular cyclisation and oxidation to produce dopachrome and L‐DOPA. Dopachrome compounds react spontaneously to form hydroxyindole molecules, which are the precursors of DHI and DHICA, and ultimately producing eumelanin pigments with the help of tyrosinase‐related protein 1 (Tyrp1) and dopachrome tautomerase (Dct), while L‐DOPA is oxidised by Tyr to produce dopaquinone (Ito and Wakamatsu 2008). As well as being an intermediate step for eu‐ and pheomelanin synthesis, L‐DOPA can also be decarboxylated to tyramine as a precursor of thyroid hormones or to dopamine and catecholamines with DDC, which decarboxylated L‐DOPA to dopamine (Slominski, Zmijewski, and Pawelek 2012). Furthermore, L‐DOPA appears to be a neurotransmitter in its own right (Misu et al. 1996). A medium supplemented with stable L‐DOPA stimulates melanocyte proliferation and Tyr activity in Cloudmann melanoma cells (Slominski, Zmijewski, and Pawelek 2012). In addition, L‐DOPA can affect neurotransmitter gene expression by binding to GPR143 (Lopez et al. 2008), which is upregulated in *MC1R*_*VI*_ owl feathers. GPR143 is a seven‐transmembrane G‐coupled receptor that causes ocular albinism when mutated in mammals (Bueschbell, Manga, and Schiedel 2022). In pigment cells, GPR143 interacts with Tyr to control melanosome maturation (Cortese et al. 2005) and with MLANA, which affects its stability (Giordano et al. 2009). GPR143 knockout murine melanocytes present reduced alpha‐MSH‐induced MITF activity and lower *PMEL* expression (Falletta et al. 2014). In addition, GPR143 forms dimers with dopamine receptors (D_2/3_R) and affects their binding affinity for dopamine (Bueschbell, Manga, and Schiedel 2022), so L‐DOPA coupled with GPR143 may affect the expression of neurotransmitter‐related genes in feather follicles. Another possibility is that L‐DOPA indirectly affects the expression of neurotransmitter‐related genes via the synthesis of dopamine and catecholamine. In 1992, Schallreuter et al. (Schallreuter et al. 1992) showed that the production of dopamine, epinephrine and norepinephrine from L‐DOPA occurs in human skin keratinocytes and melanocytes. Interestingly, epinephrine increases melanin synthesis under UVB irradiation in human cells. However, only keratinocytes are capable of synthesising epinephrine, but melanocytes have beta‐2‐adrenergic receptors that bind epinephrine (Sivamani, Porter, and Isseroff 2009). On the other hand, dopamine receptors are not known to be present in hair and feather follicles, so dopamine cannot directly regulate melanin synthesis. However, Ono et al. found pigment‐dependent thermal sensitivity in humans and mice (Ono et al. 2017), showing that the *TYRP1* mutant brown allele (loss of function) reduces pain sensitivity, establishing a link between dopaminergic signalling and melanin levels. L‐DOPA can also affect serotonin levels. Serotonin and its receptors are present in melanocytes, as is the transporter SLC6A4. However, the role of serotonin in melanogenesis is unclear (Slominski, Wortsman, and Tobin 2005).

From an evolutionary perspective, our results link melanin colouration to the expression of neurotransmitter‐related genes in the growing feathers of barn owls. Pleiotropy and linkage disequilibrium could explain the observed co‐expression of the neurotransmitter‐related genes and melanic genes in rufous *MC1R*_*VI*_ barn owls (Ducrest, Keller, and Roulin 2008; San‐Jose and Roulin 2018). In flycatchers (*Ficedula* species), linkage disequilibrium is expected at a distance of 17 kb, beyond which that it falls to background level (Kawakami et al. 2014). In barn owls, the proximity of non‐melanistic genes to melanistic genes is not the reason for co‐expression (Table S9). Furthermore, currently we have not identified any mutations linked to *MC1R* genotypes, and we cannot exclude linkage disequilibrium as a potential mechanism for such co‐expression in *MC1R*_*VI*_ barn owls (Cumer et al. 2024). Indeed, we recently discovered other regions of the genome that may also affect colour variation (Cumer et al. 2024).

In conclusion, we have shown differential co‐expression of melanic and neurotransmitter‐related genes in the growing feathers of barn owls *MC1R*_*VI*_. The potential pleiotropic roles of *MC1R*, *MITF* and L‐DOPA should be further investigated in the growing feathers of barn owls in order to at least partially explain the melanic and non‐melanic expression of genes according to *MC1R* genotypes. L‐DOPA and its derivatives offer a wider range of possibilities for explaining the co‐expression of melanin and neurotransmitter‐related genes. They regulate melanin synthesis and melanocyte homoeostasis and have neurotransmitter functions. The ability to give rise to dopamine, epinephrine and norepinephrine and to cross the blood brain barrier for L‐DOPA broadens the possibilities of regulation and calls into question the recurrent link between melanin and other phenotypic traits in the barn owl. In this species, melanic colouration is associated with sexual attributes (5‐alpha reductase expression and ovary size) (Beziers et al. 2017; Roulin 2009), resistance to oxidative stress (Roulin et al. 2011), foraging (Charter et al. 2015) and reproductive strategies (Dreiss et al. 2012; Kvalnes et al. 2022). These neurotransmitter‐related genes could provide a link to other phenotypic traits for the following reasons. First, neural crest cell‐derived melanocytes share many features with neurons: cell polarisation, dendrite formation and communication with multiple keratinocytes (Cui and Man 2023). Secondly, melanocytes may play a crucial role in communication with the central neural system via nerve endings, the hypothalamic–pituitary–adrenal (HPA) axis and act as sensory regulators in the skin (Ascsillán and Kemény 2024; Slominski, Paus, and Wortsman 1993). More recently, it has been proposed that melanocytes constitute the photosensory system of the skin (Iyengar 2013) and that opsins modulate melanin synthesis (de Assis et al. 2018; Ozdeslik et al. 2019). Third, neurotransmitters released by sympathetic nerves affect melanin deposition in mice's hair (Zhang et al. 2020), showing that the signal transmitted by the brain affects melanin levels in hair follicles. Differential expression of neurotransmitter‐related genes in the different *MC1R* genotypes may help to link barn owl feather colour to other phenotypic traits.

## Author Contributions

**Anne‐Lyse Ducrest:** conceptualization, investigation, writing – original draft, methodology, validation, visualization, writing – review and editing, formal analysis, data curation, supervision, software. **Luis M. San‐Jose:** conceptualization, investigation, writing – original draft, methodology, validation, visualization, writing – review and editing, formal analysis, supervision, resources, software. **Samuel Neuenschwander:** software, methodology, writing – review and editing, formal analysis. **Emanuel Schmid‐Siegert:** methodology, writing – review and editing, software. **Céline Simon:** methodology, writing – review and editing. **Marco Pagni:** methodology, writing – review and editing, software. **Christian Iseli:** methodology, writing – review and editing, software. **Hannes Richter:** methodology, writing – review and editing. **Nicolas Guex:** methodology, writing – review and editing, software. **Tristan Cumer:** methodology, writing – review and editing, software. **Emmanuel Beaudoing:** methodology, writing – review and editing, software. **Mélanie Dupasquier:** methodology, writing – review and editing. **Pauline Charruau:** methodology, writing – review and editing. **Pauline Ducouret:** methodology, writing – review and editing. **Ioannis Xenarios:** writing – review and editing, software, methodology, project administration. **Jérôme Goudet:** funding acquisition, resources, conceptualization, supervision, writing – review and editing, project administration. **Alexandre Roulin:** resources, funding acquisition, conceptualization, supervision, writing – review and editing, project administration.

## Conflicts of Interest

The authors declare no conflicts of interest.

## Supporting information


**Table S1.** Characteristics of the individual used for the RNA‐seq analysis.
**Table S2**. RNA sequencing statistics of mapping of the RNAseq reads in bp.
**Table S3**. New primers and probes used in the RT‐qPCR experiments, other primers and probes were described in San‐Jose et al., 2017 and Béziers et al., 2019 (Beziers et al., 2019; San‐Jose, Ducrest, et al., 2017).
**Table S4**. Results of STAR‐ HTSeq—DESeq2 differentially expressed genes between *MC1R*_*VV*_ and *MC1R*_*VI*_ males.
**Table S5**. Results of Kallisto—DESeq2 differentially expressed genes between *MC1R*_*VV*_ and *MC1R*_*VI*_ males (see xlsx file).
**Table S6**. Summary of genes found in STAR‐HTSeq‐ and Kallisto‐DESeq2 analysis.
**Table S7**. Results of the 3 trials of Kallisto‐DESeq2 differentially expressed genes between *MC1R*_*VV*_ and *MC1R*_*VI*_ males picking at random one of the 2 siblings of the 5 broods.
**Table S8**. *MC1R* genotypes derived gene significance and module membership of the WGCNA dark‐olive‐green module containing the differentially expressed genes found with Kallisto‐DESeq2 method.
**Table S9**. Location of the differentially expressed genes on barn owl scaffolds and compared to their position in the chicken (Gga6) and golden eagle (bAquChr1.4).
**Figure S1**. Co‐expression network analysis using WGCNA.

## Data Availability

The raw reads of the RNA‐Seq are available at NCBI in the BioProject: PRJNA1119676, sample accession numbers from SAMN41661779 to SAMN41661811.
