# Supplementary material for: Melanin and Neurotransmitter Signalling Genes Are Differentially Co‐Expressed in Growing Feathers of White and Rufous Barn Owls
Source: Pigment Cell Melanoma Res. 2025 Feb 5;38(2):e70001. doi: 10.1111/pcmr.70001 (PMC11799826; doi:10.1111/pcmr.70001)
Supplement: Supplementary file 1 — Table S1. Characteristics of the individual used for the RNA‐seq analysis. Table S2. RNA sequencing statistics of mapping of the RNAseq reads in bp. Table S3. New primers and probes used in the RT‐qPCR experiments, other primers and probes were described in San‐Jose et al., 2017 and Béziers et al., 2019 (Beziers et al., 2019; San‐Jose, Ducrest, et al., 2017). Table S4. Results of STAR‐ HTSeq—DESeq2 differentially expressed genes between MC1R VV and MC1R VI males. Table S5. Results of Kallisto—DESeq2 differentially expressed genes between MC1R VV and MC1R VI males (see xlsx file). Table S6. Summary of genes found in STAR‐HTSeq‐ and Kallisto‐DESeq2 analysis. Table S7. Results of the 3 trials of Kallisto‐DESeq2 differentially expressed genes between MC1R VV and MC1R VI males picking at random one of the 2 siblings of the 5 broods. Table S8. MC1R genotypes derived gene significance and module membership of the WGCNA dark‐olive‐green module containing the differentially expressed genes found with Kallisto‐DESeq2 method. Table S9. Location of the differentially expressed genes on barn owl scaffolds and compared to their position in the chicken (Gga6) and golden eagle (bAquChr1.4). Figure S1. Co‐expression network analysis using WGCNA. [file PCMR-38-0-s001.docx]

**Supplementary Table 1**: Characteristics of the individual used for the RNA-seq analysis.

| **Individual Identity** | **# Library** | **# Batch** | **# Lane** | **Age** | ***MC1R*** | **Coloration** | **Brood ID** |
| --- | --- | --- | --- | --- | --- | --- | --- |
| M022903 | 27 | 3 | 11 | 38 | *VI* | -3 | 1459 |
| M031502 | 26 | 3 | 11 | 39 | *VI* | -2.5 | 1488 |
| M031506 | 44 | 0 | 2 | 34 | *VI* | -3 | 1489 |
| M031514 | 12 | 2 | 6 | 35 | *VV* | -3 | 1490 |
| M031515 | 23 | 3 | 10 | 35 | *VV* | -4 | 1497 |
| M031519 | 21 | 3 | 9 | 39 | *VV* | -4 | 1497 |
| M032107 | 1 | 0 | 1 | 36 | *VI* | -2.5 | 1424 |
| M032108 | 43 | 5 | 16 | 33 | *VV* | -4 | 1424 |
| M032114 | 2 | 0 | 1 | 36 | *VV* | -3.5 | 1426 |
| M032115 | 3 | 0 | 1 | 34 | *VV* | -7 | 1426 |
| M032142 | 6 | 1 | 4 | 33 | *VV* | -7 | 1432 |
| M032147 | 5 | 1 | 4 | 38 | *VI* | -3 | 1428 |
| M032149 | 22 | 3 | 9 | 34 | *VV* | -7 | 1428 |
| M032227 | 34 | 4 | 13 | 34 | *VI* | -3 | 1421 |
| M032309 | 10 | 1 | 5 | 35 | *VV* | -3.5 | 1430 |
| M032311 | 11 | 1 | 6 | 33 | *VI* | -2.5 | 1445 |
| M032317 | 40 | 5 | 15 | 36 | *VV* | -6.5 | 1443 |
| M032321 | 19 | 2 | 8 | 34 | *VI* | -2.5 | 1441 |
| M032330 | 31 | 4 | 12 | 33 | *VI* | -3 | 1449 |
| M032340 | 42 | 5 | 16 | 39 | *VV* | -6.5 | 1435 |
| M032356 | 46 | 0 | 2 | 39 | *VV* | -8 | 1437 |
| M032361 | 37 | 5 | 14 | 37 | *VI* | -3.5 | 1446 |
| M032368 | 36 | 5 | 14 | 34 | *VV* | -3 | 1453 |
| M032370 | 32 | 4 | 13 | 38 | *VV* | -3.5 | 1453 |
| M032374 | 16 | 2 | 7 | 37 | *VI* | -4 | 1468 |
| M032378 | 39 | 5 | 15 | 32 | *VI* | -2 | 1466 |
| M032431 | 17 | 2 | 8 | 35 | *VV* | -6.5 | 1465 |
| M032436 | 24 | 3 | 10 | 36 | *VI* | -3.5 | 1471 |
| M032441 | 35 | 4 | 14 | 40 | *VV* | -7 | 1477 |
| M032445 | 47 | 0 | 3 | 38 | *VV* | -3 | 1470 |
| M032454 | 28 | 4 | 11 | 38 | *VV* | -4 | 1474 |
| M032473 | 25 | 3 | 10 | 34 | *VV* | -6.5 | 1482 |
| M032494 | 13 | 2 | 6 | 35 | *VI* | -4 | 1498 |

# Libraires: number of the library; # Batch: Library preparation series; # Lane: Illumina sequencing lane; Age: Age in days of the nestlings when the feather was plucked; *MC1R*: genotype of the *MC1R* gene according to the site V126I; Coloration: visual measure of the coloration of the breast feathers: from -8: = white; to -1: reddish {San-Jose, 2015 #11864} and Brood_ID: the identity of the brood in which the nestling is born.

**Supplementary Table 2**: RNA sequencing statistics of mapping of the RNAseq reads in bp.

| **Library** | **Raw reads** | **>=Q30** | **% GC** | **Cleaned reads** | **Uniquely mapped** | **Multiple mapped** | **unmapped** | **% uniquely mapped** | **% mapping rate** |
| --- | --- | --- | --- | --- | --- | --- | --- | --- | --- |
| 1 | 64 286 713 | 92.3 | 53.0 | 63 239 793 | 39 149 271 | 14 749 035 | 9 341 487 | 61.9 | 85.2 |
| 2 | 51 578 326 | 91.7 | 52.5 | 50 551 286 | 29 823 724 | 14 323 485 | 6 404 077 | 59.0 | 87.3 |
| 3 | 66 220 096 | 92.0 | 51.5 | 64 253 822 | 37 798 006 | 18 031 510 | 8 424 306 | 58.8 | 86.9 |
| 5 | 72 644 913 | 93.4 | 53.0 | 71 374 775 | 40 591 233 | 22 106 576 | 8 676 966 | 56.9 | 87.8 |
| 6 | 69 863 823 | 93.2 | 53.0 | 68 361 700 | 39 980 683 | 17 807 177 | 10 573 840 | 58.5 | 84.5 |
| 10 | 79 731 211 | 92.8 | 53.0 | 78 230 990 | 46 119 129 | 23 510 744 | 8 601 117 | 59.0 | 89.0 |
| 11 | 83 032 694 | 92.0 | 53.0 | 81 570 977 | 45 015 756 | 26 459 155 | 10 096 066 | 55.2 | 87.6 |
| 12 | 52 034 627 | 93.3 | 53.0 | 51 224 473 | 30 384 108 | 14 698 881 | 6 141 484 | 59.3 | 88.0 |
| 13 | 73 638 073 | 93.2 | 54.0 | 72 573 391 | 40 937 629 | 22 195 452 | 9 440 310 | 56.4 | 87.0 |
| 16 | 65 053 962 | 92.1 | 52.5 | 63 420 385 | 37 588 950 | 17 680 903 | 8 150 532 | 59.3 | 87.1 |
| 17 | 76 073 179 | 92.3 | 52.5 | 74 465 372 | 42 643 587 | 22 727 061 | 9 094 724 | 57.3 | 87.8 |
| 19 | 72 287 647 | 91.9 | 52.5 | 70 646 889 | 39 475 845 | 21 324 639 | 9 846 405 | 55.9 | 86.1 |
| 21 | 91 541 796 | 91.5 | 52.0 | 89 809 084 | 52 383 115 | 25 711 755 | 11 714 214 | 58.3 | 87.0 |
| 22 | 65 522 070 | 93.3 | 54.0 | 64 774 228 | 37 583 680 | 18 668 905 | 8 521 643 | 58.0 | 86.8 |
| 23 | 79 560 041 | 92.7 | 54.0 | 78 503 905 | 47 105 156 | 22 131 156 | 9 267 593 | 60.0 | 88.2 |
| 24 | 79 421 933 | 93.5 | 55.5 | 78 654 726 | 44 899 023 | 22 705 816 | 11 049 887 | 57.1 | 86.0 |
| 25 | 67 802 759 | 93.2 | 54.0 | 66 902 494 | 36 005 378 | 22 008 107 | 8 889 009 | 53.8 | 86.7 |
| 26 | 71 767 799 | 93.3 | 55.0 | 70 854 744 | 39 457 359 | 20 975 379 | 10 422 006 | 55.7 | 85.3 |
| 27 | 80 800 525 | 93.2 | 55.5 | 79 950 135 | 43 811 833 | 24 966 190 | 11 172 112 | 54.8 | 86.0 |
| 28 | 69 208 862 | 92.8 | 55.5 | 68 350 857 | 34 678 962 | 24 035 318 | 9 636 577 | 50.7 | 85.9 |
| 31 | 88 262 193 | 92.6 | 53.5 | 86 994 038 | 48 189 575 | 27 845 333 | 10 959 130 | 55.4 | 87.4 |
| 32 | 83 378 401 | 92.9 | 53.5 | 83 378 401 | 48 036 303 | 22 417 735 | 12 924 363 | 57.6 | 84.5 |
| 34 | 77 639 318 | 91.3 | 53.0 | 76 335 001 | 43 898 661 | 20 866 930 | 11 569 410 | 57.5 | 84.8 |
| 35 | 63 236 911 | 91.1 | 52.0 | 61 864 161 | 36 659 087 | 17 248 031 | 7 957 043 | 59.3 | 87.1 |
| 36 | 87 405 621 | 91.5 | 52.5 | 87 405 621 | 49 018 902 | 25 309 056 | 13 077 663 | 56.1 | 85.0 |
| 37 | 88 254 133 | 90.8 | 52.0 | 86 708 020 | 49 109 276 | 27 038 087 | 10 560 657 | 56.6 | 87.8 |
| 39 | 75 206 127 | 90.4 | 52.5 | 73 916 160 | 39 083 709 | 25 503 780 | 9 328 671 | 52.9 | 87.4 |
| 40 | 85 012 161 | 90.4 | 52.5 | 83 436 301 | 46 354 209 | 24 234 160 | 12 847 932 | 55.6 | 84.6 |
| 42 | 84 555 476 | 90.6 | 51.5 | 82 888 970 | 49 611 007 | 23 186 354 | 10 091 609 | 59.9 | 87.8 |
| 43 | 83 080 716 | 89.6 | 50.5 | 80 875 271 | 52 141 317 | 16 865 376 | 11 868 578 | 64.5 | 85.3 |
| 44 | 89 004 629 | 90.6 | 53.0 | 87 849 753 | 49 451 834 | 27 234 225 | 11 163 694 | 56.3 | 87.3 |
| 46 | 76 611 353 | 89.8 | 52.0 | 75 056 214 | 42 864 398 | 22 019 857 | 10 171 959 | 57.1 | 86.4 |
| 47 | 83 134 382 | 90.6 | 52.5 | 81 551 313 | 46 667 733 | 23 924 104 | 10 959 476 | 57.2 | 86.6 |

>=Q30: percent of raw reads with a base quality score above 30, % GC: percent of GC of raw reads.

**Supplementary table 3**: New primers and probes used in the RT-qPCR experiments, other primers and probes were described in San-Jose *et al*., 2017 and Béziers *et al*., 2019 {Beziers, 2019 #14065;San-Jose, 2017 #12204}.

| **Primer** | | | |
| --- | --- | --- | --- |
| **Name** | **Sequence** | **ec* (nM)** | **PCR efficiency** |
| CALB1_237Fw | GCTTGCTCAGATATTACCAACA | 300 |  |
| CALB1_330Rv | TCTCCATGTCTGCATGAAATC | 150 | 99.0 |
| CALB1_269FAMQ1 | TCTCCATGTCTGCATGAAATC | 300 |  |
| DDC_1044Fw | ATTATCGGCACTGGCAAATC | 150 |  |
| DDC_1126Rv | CCATACATCCTCAGCACGAA | 150 | 99.8 |
| DDC_1076FAMQ1 | GAGGTTCCGCTCCCTGAAGCTCTG | 150 |  |
| FDNC9L_-98Fw | CAGCTTCTCAGCTACCTCC | 300 |  |
| FDNC9L_-18Rv | TCGGGTGGTTTCTTCTCTC | 150 | 98.8 |
| FDNC9L_-71FAMQ1 | CAAACATCTTCCCTTTCCACGGCGAG | 300 |  |
| GPR143_618Fw | GGTCAACCCAATCCTGTTTA | 300 |  |
| GPR143_773Rv | GACGACCAACAAATAATGAATACC | 300 | 100.2 |
| GPR143_640FAMQ1 | AGGACAGTATCAGCAGTTGCCTCC | 300 |  |
| MFSD12_492Fw | TCGGAACCTGTCCCTCATTGT | 300 |  |
| MFSD12_576Rv | CGCGTATGGCTTCTCTTTGGT | 150 | 100.4 |
| MFSD12_519FAMQ1 | GCTGGGGGTCGTGTTCTCCTTCATCT | 300 |  |
| RAB38_157Fw | AGCTGGGATGCTGAGACTGT | 300 |  |
| RAB38_303Rv | TGCTTCAAATGTTGCAGGTC | 150 | 100.6 |
| RAB38_247FAMQ1 | GAGGCCATGGGGGCATTTATTGTC | 300 |  |
| SLC6A4_942Fw | GGTGTCCTCTACTATGTGAAAC | 300 |  |
| SLC6A4_1130Rv | GGCATCTTGGTAGCAGTTG | 300 | 99.8 |
| SLC6A4_978FAMQ1 | CTCCTTGCCACCGAGGTTTGGG | 150 |  |
| SYNGR3_400Fw | GGCTTTTCAGGTTTCTTGTC | 300 |  |
| SYNGR3_553Rv | CCCAGACGATGATGGAGA | 150 | 99.9 |
| SYNGR3_425FAMQ1 | TGTGGTTTGTAGCCTTCTGCTTCCT | 300 |  |
| TBXA2R_812Fw | CCCTCCTGATCTTCATCGTC | 300 |  |
| TBXA2R_908Rv | CAGCATCTTCTGCGTCTCTG | 150 | 98.9 |
| TBXA2R_842FAMQ1 | CTGCAGCAGCTCCCAGCCAAC | 300 |  |
| VAT1L_1082Fw | TCTGGAAGAGGTAAAGGAGG | 100 |  |
| VAT1L_1244Rv | CTCGTTGTCCCCTTCATG | 100 | 99.9 |
| VAT1L_1195FAMQ1 | TCCTCTTCTCCAGCCTCACTTGTCT | 150 |  |
| VIP_242Fw | CCAGAATTATTGATAGCTCCCA | 150 |  |
| VIP_530Rv | GTTCCGCTTCATCTCGAA | 300 | 100.0 |
| VIP_389FAMQ1 | CGCCACTCTGACGCTGTCTTCAC | 300 |  |

*ec: end concentration

**Supplementary Table 4:** Results of STAR- HTSeq - Deseq2 differentially expressed genes between *MC1R_VV_* and *MC1R_VI_* males.

(see csv file: https://github.com/alroulinn/RNA-Seq)

**Supplementary Table 5:** Results of Kallisto - Deseq2 differentially expressed genes between *MC1R_VV_* and *MC1R_VI_* males.

(see csv file: <https://github.com/alroulinn/RNA-Seq>)

**Supplementary Table 6:** Summary of genes found in STAR-HTSeq- and Kallisto-DESeq2 analysis.

|  | **DESeq2-STAR-HTSeq** | | **DESeq2_Kallisto** | |
| --- | --- | --- | --- | --- |
| **Genes** | **LFC** | **FDR** | **LFC** | **FDR** |
| ***CALB1*** | 2.56 | 4.76E-06 | 2.5 | 5.97E-06 |
| *CDK15* | NA | NA | -1.0 | 3.17E-02 |
| ***DDC*** | -2.01 | 2.28E-04 | -1.9 | 2.47E-04 |
| *FLO11/PTX2/LOC116960306* | -0.93 | 1.35E-02 | -0.9 | 1.49E-02 |
| *FNDC9-l/LOC104357634* | -1.91 | 1.68E-03 | -1.9 | 2.04E-03 |
| *FOSB* | NA | NA | -1.8 | 3.17E-02 |
| *GPR143* | -1.44 | 1.83E-03 | -1.4 | 2.04E-03 |
| *KLRB1/LOC116960911* | -1.00 | 7.00E-03 | -1.0 | 6.73E-03 |
| *MFSD12* | -0.73 | 8.07E-05 | -0.7 | 7.58E-05 |
| *MLANA* | -1.29 | 9.79E-04 | -1.3 | 8.57E-04 |
| *PFDN2/LOC122152587* | NA | NA | -4.6 | 6.10E-06 |
| *PMEL* | -1.47 | 2.67E-04 | -1.5 | 2.54E-04 |
| *RAB38* | -1.18 | 1.03E-02 | -1.2 | 1.18E-02 |
| *RAB3C* | -1.41 | 3.18E-02 | -1.4 | 3.17E-02 |
| *RASL10B* | -1.67 | 8.93E-03 | -1.5 | 1.33E-02 |
| *SERPINE3* | -1.17 | 2.03E-02 | NA | NA |
| ***SLC28A2 / LOC104360559*** | NA | NA | -1.0 | 4.00E-02 |
| *SLC45A2* | -3.71 | 3.96E-07 | -3.7 | 4.59E-07 |
| ***SLC6A4*** | -1.43 | 1.57E-04 | -1.4 | 1.55E-04 |
| *STK32A* | -0.91 | 3.18E-02 | NA | NA |
| ***SYNGR3*** | -1.17 | 6.60E-03 | -1.2 | 7.29E-03 |
| *TBXA2R* | -0.75 | 1.50E-02 | -0.8 | 1.31E-02 |
| *~~TRMT12~~* | ~~-0.36~~ | ~~4.95E-02~~ | *~~-0.4~~* | *~~3.17E-02~~* |
| *TYR* | -3.65 | 3.96E-07 | -3.6 | 4.59E-07 |
| *TYRP1* | -6.94 | 2.31E-07 | -6.8 | 4.59E-07 |
| ***VAT1L*** | -1.15 | 4.86E-03 | -1.2 | 4.75E-03 |
| ***VIP/LOC104368820/LOC122153719*** | -2.03 | 1.44E-03 | -2.5 | 3.49E-02 |
| ***VSTM2L*** | -0.84 | 2.92E-02 | -0.8 | 2.24E-02 |

^1^LFC: log2 fold change, ^2^FDR: FDR-corrected p-value.

In red genes involved in melanogenesis, in bold neurotransmitter-related genes. *TRMT12* was not considered as the log fold change is 1.3x change in expression.

**Supplementary Table 7:** Results of the 3 trials of Kallisto-DESeq2 differentially expressed genes between *MC1R_VV_* and *MC1R_VI_* males picking at random one of the 2 siblings of the 5 broods.

|  | Removed libraries | | | | | |
| --- | --- | --- | --- | --- | --- | --- |
|  | 1, 2, 5, 22, 36 | | 1,3,22,23,32 | | 3,21,22,36,43 | |
| Genes | LFC1 | padj1 | LFC2 | padj2 | LFC3 | padj3 |
| *CALB1* | 2.21 | 0.0019 | 2.48 | 0.0003 | 2.52 | 0.00017 |
| *CDK15* | -1.06 | 0.0213 | NA | NA | NA | NA |
| *DDC* | -1.93 | 0.0019 | -1.89 | 0.0119 | -1.86 | 0.00608 |
| *FLO11/PTX2/LOC116960306* | -1.00 | 0.0237 | -1.06 | 0.0119 | -0.96 | 0.03508 |
| *FNDC9-L/LOC104357634* | -1.94 | 0.0023 | -2.07 | 0.0029 | -1.93 | 0.01634 |
| *FOSB* | NA | NA | -2.10 | 0.0144 | NA | NA |
| *GPR143* | -1.48 | 0.0096 | -1.54 | 0.0250 | -1.39 | 0.02466 |
| *HBE1/LOC116963251* | NA | NA | -1.91 | 0.0101 | NA | NA |
| *KLRB1/LOC116960911* | -0.98 | 0.0495 | -1.05 | 0.0104 | -1.02 | 0.01798 |
| *MFSD12* | -0.73 | 0.0012 | -0.73 | 0.0026 | -0.70 | 0.00308 |
| *MLANA* | -1.27 | 0.0073 | -1.25 | 0.0156 | -1.21 | 0.02615 |
| *PFDN2/LOC122152587* | -4.54 | 0.0004 | -4.71 | 0.0002 | -5.05 | 0.00002 |
| *PMEL* | -1.49 | 0.0023 | -1.51 | 0.0119 | -1.46 | 0.00356 |
| *RAB38* | -1.24 | 0.0204 | NA | NA | NA | NA |
| *RASL10B* | -1.56 | 0.0204 | NA | NA | NA | NA |
| *SLC28A2/LOC104360559* | -1.10 | 0.0189 | NA | NA | NA | NA |
| *SLC45A2* | -3.84 | 0.0000 | -3.64 | 0.0000 | -3.41 | 0.00017 |
| *SLC6A4* | -1.42 | 0.0022 | -1.36 | 0.0104 | -1.35 | 0.00615 |
| *SORCS2* | 0.78 | 0.0113 | NA | NA | NA | NA |
| *SYNGR3* | -1.16 | 0.0204 | NA | NA | NA | NA |
| *TRMT12* | *-0.46* | *0.0004* | *-0.49* | *0.0000* | *-0.39* | *0.02732* |
| *TYR* | -3.72 | 0.0000 | -3.56 | 0.0000 | -3.37 | 0.00017 |
| *TYRP1* | -5.98 | 0.0001 | -6.41 | 0.0001 | -6.02 | 0.00017 |
| *VAT1L* | -1.18 | 0.0136 | -1.22 | 0.0145 | -1.15 | 0.03508 |
| *VIP/LOC104368820* | -2.59 | 0.0189 | NA | NA | NA | NA |

LFC: log fold change, padj: FDR adjusted p values, 1,2,3: the 3 different trials.

**Supplementary Table 8:** *MC1R* genotypes derived gene significance and module membership of the WGCNA dark-olive-green module containing the differentially expressed genes found with Kallisto-DESeq2 method.

| **Genes** | **Module Membership** | **Gene Significance** |
| --- | --- | --- |
| *ADD2* | 0.618 | -0.384 |
| *AMPD1* | 0.493 | -0.485 |
| *ANK1* | 0.649 | -0.435 |
| *ART4* | 0.762 | -0.754 |
| *CUNH15orf48* | 0.643 | -0.448 |
| *DMTN* | 0.884 | -0.708 |
| *GP1BB* | 0.812 | -0.831 |
| *GP9* | 0.591 | -0.160 |
| *LOC104355943/ SLC7A2* | 0.797 | -0.857 |
| *LOC104358558/ CA1* | 0.559 | -0.323 |
| *LOC104359698/ HBB* | 0.896 | -0.791 |
| *LOC104360120/ PCTP* | 0.941 | -0.866 |
| *LOC104360184/ histone H5* | 0.897 | -0.660 |
| *LOC104368414/ TRIM27* | 0.781 | -0.780 |
| *LOC104369299/ thaicobrin-like* | 0.786 | -0.225 |
| *LOC104369464/ MISP* | 0.696 | -0.882 |
| *LOC116959343/ HBA1* | 0.919 | -0.789 |
| *LOC116961035/ HBA2* | 0.874 | -0.844 |
| *LOC116961333/ MENTL* | 0.527 | -0.708 |
| *LOC116962070* | 0.728 | -0.434 |
| *LOC116963251/ HBE1* | 0.639 | -0.901 |
| *LOC116963252/ HBBL* | 0.766 | -0.727 |
| *LOC116963855* | 0.925 | -0.910 |
| *LOC116965363* | 0.421 | -0.033 |
| *LOC122152617/ TMOD4L* | 0.705 | -0.581 |
| *LOC122153234/ SLC7A2L* | 0.815 | -1.125 |
| *LOC122153235/ SLC7A2L* | 0.659 | -0.645 |
| *LOC122154907* | -0.512 | 1.417 |
| *NOS2* | 0.408 | -0.264 |
| *RFESD* | 0.720 | -0.399 |
| *RHAG* | 0.891 | -0.835 |
| *RLBP1* | 0.554 | -0.920 |
| *SLC46A3* | 0.683 | -0.664 |
| *SLC4A1* | 0.863 | -0.806 |
| *TAL1* | 0.778 | -0.316 |
| *TESPA1* | 0.627 | -0.844 |
| *WDR72* | 0.554 | -0.292 |

Gene trait significance corresponds to the Cohen’s D effect size coefficient, the Module Membership was calculated with Spearman Rho correlation coefficient.

**Supplementary Table 9:** Location of the differentially expressed genes on the barn owl scaffolds and compared to their position in chicken (Gga6) and golden eagle (bAquChr1.4)

|  | **Barn owl** | | |  | **Chicken** | | **Golden eagle** | |
| --- | --- | --- | --- | --- | --- | --- | --- | --- |
| **Genes** | **Scaffold name** | **Start position** | **Stop position** | **Distance between two consecutive genes (bp)** | **Chs*** | **position** | **Chs** | **position** |
| ***KLRB1/LOC116960911*** | **Super-Scaffold_**  **100000100064** | 279,735 | 283,468 |  |  | not found |  | not found |
| ***OCA2*** | **Super-Scaffold_14** | 1 873 622 | 2 057 732 | 254 947 | **1** | 131,975,543-132,105,662 | **23** | 10,148,333-10,331,085 |
| ***FNDC9L*** | **Super-Scaffold_14** | 2 312 679 | 2 315 615 | 6 075 000 | **1** | 131,722,616-131,727,023 | **23** | 10,584,414-10,585,684 |
| ***GPR143*** | **Super-Scaffold_14** | 8 377 386 | 8 390 615 |  | **1** | 126518232-126531997 | **23** | 16587049-16601228 |
| ***SLC6A4*** | **Super-Scaffold_19** | 5 343 919 | 5 356 547 | **6 869 079** | **19** | 6,349,841-6,361,974 | **10** | 38,549,504-38,571,471 |
| ***RASL10B*** | **Super-Scaffold_19** | 12 225 626 | 12 235 670 |  | **19** | 4,534,259-4,537,686 | **10** | 36189473-36194140 |
| ***RAB38*** | **Super-Scaffold_2** | 9 399 350 | 9 420 015 | 398 918 | **1** | 189,576,307-189,596,533 | **19** | 19,035,707-19,055,479 |
| ***TYR*** | **Super-Scaffold_2** | 9 818 933 | 9 865 310 | 23 317 273 | **1** | 189,153,122-189,202,896 | **19** | 18,583,951-18,634,903 |
| ***SOX10*** | **Super-Scaffold_2** | 33 182 583 | 33 192 228 |  | **1** | 51,055,215-51,064,410 | **17** | 2,287,363-2,298,306 |
| ***MFSD12*** | **Super-Scaffold_**  **20000042** | 15 266 436 | 15 277 241 | 11 754 | **28** | 1,155,041-1,161,700 | **12** | 5,621,045-5,632,208 |
| ***TBXA2R*** | **Super-Scaffold_**  **20000042** | 15 288 995 | 15 290 557 |  | **28** | 1,140,092-1,145,539 | **12** | 5,643,602-5,648,782 |
| ***PCSK2*** | **Super-Scaffold_22** | 3 157 428 | 3 262 578 | **18 930 768** | **3** | 12,111,551-12,286,838 | **8** | 29,309,098-29,437,742 |
| ***VIP*** | **Super-Scaffold_22** | 22 193 346 | 22 200 105 |  | **3** | 49,586,424-49,602,336 | **8** | 15,367,473-15,374,422 |
| ***SLC7A11*** | **Super-Scaffold_23** | 12 965 295 | 13 025 443 | 15 007 180 | **4** | 28,905,344-28,960,060 | **1** | 71,773,818-71,834,032 |
| ***PMEL*** | **Super-Scaffold_23** | 28 032 623 | 28 035 446 |  | **33** | 7,092,009-7,095,774 | **15** | 30,381,706-30,385,768 |
| ***AGRP*** | **Super-Scaffold_26** | 1 923 458 | 1 924 969 | 14 593 242 | **11** | 1,397,797-1,400,275 | **9** | 22,377,393-22,378,764 |
| ***VAT1L*** | **Super-Scaffold_26** | 16 518 211 | 16 556 414 | **5 964 906** | **11** | 13,982,142-14,025,331 | **9** | 7,378,422-7,441,409 |
| ***MC1R*** | **Super-Scaffold_26** | 22 521 320 | 22 522 632 |  | **11** | 18,840,387-18,841,759 | **9** | 1,440,185-1,441,123 |
| ***DDC*** | Super-Scaffold_27 | 32 254 616 | 32 316 315 |  | **2** | 80755118-80807342 | **4** | 76341592-76417028 |
| ***SYNGR3*** | **Super-Scaffold_28** | 1 998 211 | 2 007 261 |  | **14** | 6,572,269-6,596,578 | **25** | 1,877,502-1,898,288 |
| ***MITF*** | **Super-Scaffold_3** | 14 428 241 | 14 538 240 |  | **12** | 15,882,966-15,968,266 | **20** | 19,102,072-19,213,155 |
| ***ASIP*** | **Super-Scaffold_33** | 2 058 216 | 2 095 350 | **15 422 927** | **20** | 1,567,219-1,596,889 | **3** | 16,017,224-16,024,395 |
| ***VSTM2L*** | Super-Scaffold_33 | 17 518 277 | 17 533 478 |  | **20** | 10415519-10422917 | 3 | 601906-618825 |
| ***POMC*** | **Super-Scaffold_40** | 49 700 783 | 49 706 535 |  | **3** | 105,372,181-105,388,173 | **15** | 1,577,719-1,579,111 |
| ***SLC45A2*** | **Super-Scaffold_42** | 16 186 179 | 16 201 609 | 8 011 029 | **Z** | 10,331,208-10,346,031 | **Z** | 74,972,659-74,987,805 |
| ***RAB3C*** | **Super-Scaffold_42** | 24 212 638 | 24 351 851 | 10 188 159 | **Z** | 18063446-18207759 | **Z** | 66693509-66817153 |
| ***MLANA*** | **Super-Scaffold_42** | 34 540 010 | 34 546 433 | 3 036 834 | **Z** | 27,814,456-27,819,700 | **Z** | 56,188,006-56,194,517 |
| ***TYRP1*** | **Super-Scaffold_42** | 37 583 267 | 37 594 131 |  | **Z** | 30,823,102-30,833,822 | **Z** | 53,069,281-53,079,481 |
| ***DCT*** | **Super-Scaffold_45** | 27 504 525 | 27 523 114 |  | **1** | 147,901,713-147,921,618 | **14** | 6,856,944-6,874,906 |
| ***CPE*** | **Super-Scaffold_6** | 4 103 332 | 4 160 370 | 21 521 521 | **4** | 23,889,262-23,935,926 | **1** | 44,649,356-44,710,247 |
| ***KIT*** | **Super-Scaffold_6** | 25 681 891 | 25 736 721 | 21 001 893 | **4** | 65,269,770-65,321,836 | **1** | 30,971,102-31,011,024 |
| ***FLO11/LOC116960306*** | **Super-Scaffold_6** | 46 738 614 | 46 755 528 |  |  | not found |  | not found |
| ***CALB1*** | **Super-Scaffold_9** | 27 892 038 | 27 910 071 |  | **2** | 124,313,161-124,331,362 | **4** | 28,152,304-28,170,012 |

*Chs: chromosome; In red are melanic genes, in violet melanocortin genes. The same coloration represents genes on the same scaffold.

**Supplementary Figure 1:** Co-expression network analysis using WGCNA. a) Suitable power selection for gene clustering: mean of independence and connectivity, b) Gene clustering tree obtained by hierarchical clustering of adjacency-based dissimilarity. The colored bars below represent the modules identified using the dynamic tree cut method and merged module. c) Modules association with *MC1R* genotypes. The significant correlated modules (sky-blue and dark-olive-green are highlighted with a star). d) Relationship between skyblue module and *MC1R* genotypes.
